# Measurement of jugular foramen diameter using MRI in multiple sclerosis patients compared to control subjects

**DOI:** 10.1186/s41747-017-0008-3

**Published:** 2017-06-29

**Authors:** Giacomo Davide Edoardo Papini, Giovanni Di Leo, Moreno Zanardo, Maria Paola Fedeli, Ilaria Merli, Francesco Sardanelli

**Affiliations:** 1 Radiology Unit, IRCCS Policlinico San Donato, Via Morandi 30, 20097 San Donato Milanese, Italy; 2 PhD Course in Integrative Biomedical Research, Università degli Studi di Milano, Via Mangiagalli 31, 20133 Milano, Italy; 3 Scuola di Specializzazione in Radiodiagnostica, Università degli Studi di Milano, via Festa del Perdono 7, 20122 Milano, Italy; 4 Dipartimento di Scienze Biomediche per la Salute, Università degli Studi di Milano, Via Morandi 30, 20097 San Donato Milanese, Italy

**Keywords:** Chronic cerebrospinal venous insufficiency (CCSVI), Jugular foramina, Jugular veins, Magnetic resonance imaging (MRI), Multiple sclerosis (MS)

## Abstract

**Background:**

Multiple sclerosis (MS) is a chronic disease of the central nervous system. As an association between MS and reduced cerebral venous blood drainage was hypothesised, our aim was to compare the size of the jugular foramina in patients with MS and in control subjects.

**Methods:**

Ethics committee approval was received for this retrospective case–control study. We collected imaging and clinical data of 53 patients with MS (23 men, mean age 45 ± 9 years) and an age/gender-matched control group of 53 patients without MS (23 men, mean age 46 ± 10 years). The minimal diameter of both jugular foramina was measured on T1-weighted contrast-enhanced axial magnetic resonance images; the two diameters were summed. Student *t* test and Spearman correlation coefficient were used for analysis. Reproducibility was estimated using the Bland–Altman method.

**Results:**

The mean diameter of the right foramen in patients with MS (6.3 ± 1.6 mm) was 10% smaller than that of the controls (7.0 ± 1.4 mm) (*p* = 0.020); the mean diameter of the left foramen in patients with MS (5.6 ± 1.3 mm) was 7% smaller than that of the controls (6.0 ± 1.3 mm) (*p* = 0.089). The sum of the diameters of both jugular foramina in patients with MS (mean 11.9 ± 2.3 mm) was 8% smaller (*p* = 0.009) than that of the controls (mean 13.0 ± 2.1 mm). The differences in diameters between patients with relapsing-remitting MS and patients with secondary progressive MS were not significant (*p* ≥ 0.332). There was no significant correlation between foramen diameters and the expanded disability status scale (*p* ≥ 0.079). Intra-reader and inter-reader reproducibility were 91% and 88%, respectively.

**Conclusions:**

Jugular foramen diameter in patients with MS was 7-10% smaller than that in controls, regardless of the MS disease course.

## Key points

- The right foramen diameter was 10% smaller in MS patients compared to controls
- There was a similar difference (8%) in the sum of the diameters of both foramina in MS patients compared to controls
- The disease course of MS (relapsing–remitting or secondary progressive) was not associated with the foramen diameter
- The foramen diameter was not correlated with the expanded disability status scale

## Background

Multiple sclerosis (MS) is a chronic, autoimmune, inflammatory disease of the central nervous system characterised by inflammation, demyelination, axonal loss, and eventual neurodegeneration [1]. A number of pathophysiologic mechanisms have been advocated and the unpredictable course of the disease does not allow for anticipating whether the favourable effects of short-term treatments will be sustained [2]. Despite previous research, the pathogenesis of MS continues to challenge investigators.

Several studies have reported vascular abnormalities in patients with MS. Imaging studies have also suggested decreased cerebral perfusion, affecting areas of white matter that appears normal [3]. The main intracranial veins and venous sinuses converge to form major dural sinuses, the transverse sinus and the sigmoid sinus, which drain into the extracranial veins through the jugular foramina. Jugular foramina diameters typically range from 10 to 15 mm; the right foramen is usually larger than the left one [4]. They are composed of two parts: the *pars nervosa*, containing the glossopharyngeal nerve and the inferior petrosal sinus, and the *pars vascularis*, containing the internal jugular vein, the vagal nerve, and the spinal accessory nerve.

In our clinical experience, we have noted cases of small jugular foramina in patients with MS. Thus, the aim of this study was to measure the venous portion of the jugular foramina in a cohort of patients with MS and to compare to control subjects.

## Methods

### Study design and participants

This retrospective case–control study was approved by the local ethics committee (*Azienda Sanitaria Locale Milano 2*, Melegnano, Milan, Italy). We contacted a non-profit association of patients with MS. This association provided us with contrast-enhanced magnetic resonance imaging (MRI) examinations of 53 patients with MS (23 men and 30 women, mean age 45 ± 9 years). Because these patients have had multiple MRI studies at multiple centres, we analysed the most recent MRI examination. All patients signed an informed consent and filled out a questionnaire requesting clinical data. In particular, information on the disease course (relapsing–remitting, primary progressive, or secondary progressive) and the expanded disability status scale (EDSS) were obtained. Patient characteristics are shown in Table 1.

**Table 1:** Clinical characteristics of patients with multiple sclerosis

Characteristic	Value
Number of patients	53
Age^a^ (years)	45 ± 9
Disease course (number of patients)	
Relapsing–remitting	39
Secondary progressive	11
Primary progressive	2
Not defined	1
Age at diagnosis^a^ (years)	
Overall	33 ± 8
Relapsing–remitting	33 ± 8
Secondary progressive	34 ± 8
Primary progressive	33 and 49
Body mass index^a^ (kg/m^2^)	24 ± 4
Expanded disability status scale	
Median	2.0
25^th^ percentile	1.5
75^th^ percentile	4.5
Minimum	1
Maximum	7

^a^Mean ± standard deviation

A control group matched for age and gender was set up including a series of 53 consecutive outpatients without MS (23 men and 30 women, mean age 46 ± 10 years) who were referred to our institution for a contrast-enhanced MRI of the brain. Indications in control subjects were as follows: headache (n = 18), non-brain cancer staging (n = 5), dizziness (n = 5), hearing or visual disorders (n = 5), stroke (n = 5), primitive brain tumor (n = 4), infective diseases (n = 3), epilepsy (n = 2), and other indications (n = 6).

### Measurements

To avoid bias, an independent reader created a database pooling and randomizing the order of presentation of patients with MS and control subjects. This reader extracted from the full MRI examination only the axial contrast-enhanced T1-weighted turbo spin-echo sequence. Moreover, to avoid the identification of lesions in the white matter, this reader selected only the slices containing the jugular foramina, and verified the absence of lesions in these slices. All images were anonymised and presented to a second reader.

**Fig. 1:**
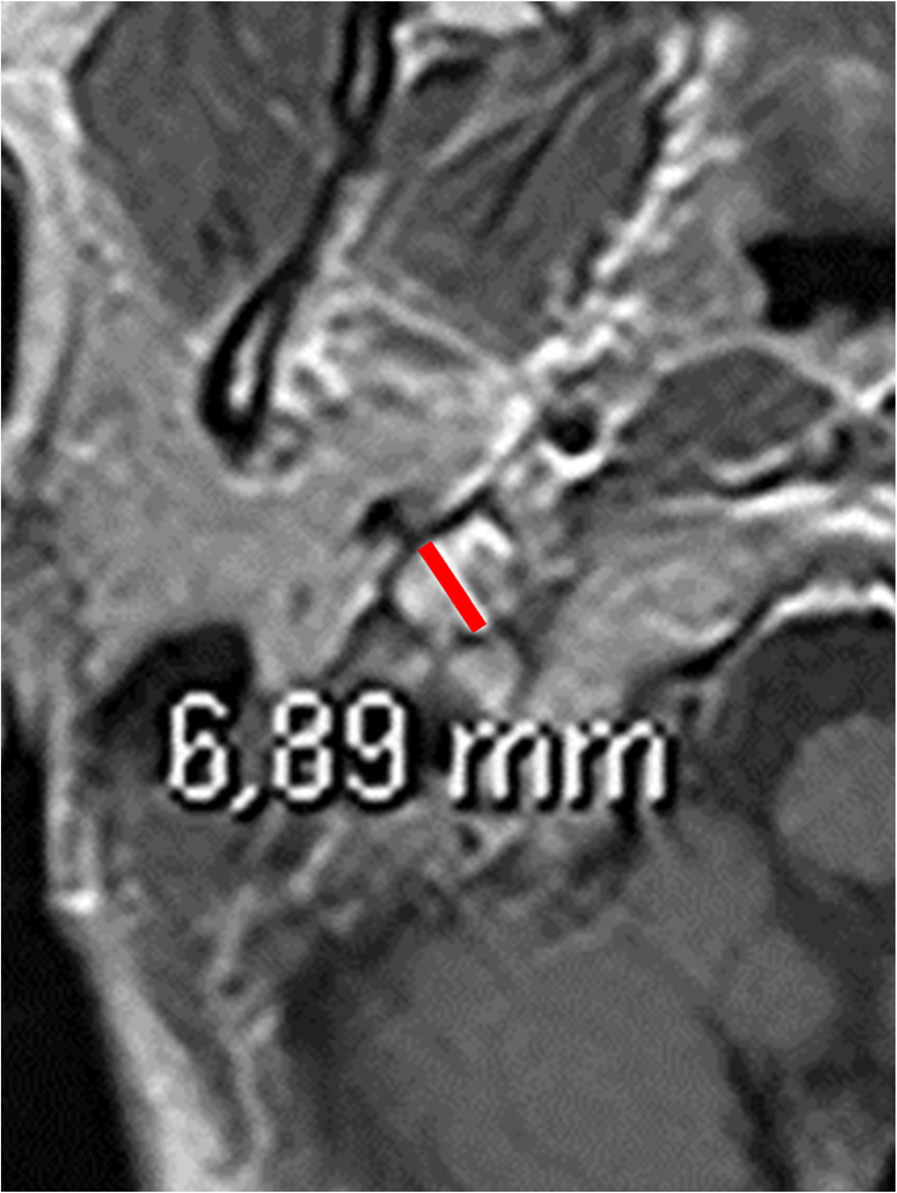
Magnification of a contrast-enhanced T1-weighted axial magnetic resonance image showing the method used to measure the jugular foramen diameter. Here the *red* caliper indicates the minimal diameter of the right jugular foramen

This second reader (who had 5 years of experience in brain MRI) measured the size of both jugular foramina. Considering the tortuosity of the foramen and its well-defined vascular portion on contrast-enhanced images, the reader measured the minimal lumen diameter of the venous portion passing through the skull base (Fig. 1). To estimate intra-reader and inter-reader reproducibility, this reader repeated the measurements after 2 weeks in a subset of 28 subjects randomly selected from the entire database, whereas a third independent reader, who had 1 year of experience, performed the measurement once for the same 28 subjects.

All images were analysed using a remote workstation (Leonardo, Siemens Medical Solutions, Erlangen, Germany) and the Syngo-Argus software (v. VE32B, Siemens Medical Solutions, Erlangen, Germany).

### Statistical analysis

Continuous data were shown as mean ± standard deviation or median and interquartile range, as appropriate. The Shapiro–Wilk test for normality of distributions was performed. Accordingly, comparisons were performed using the Student *t* test or the non-parametric Mann–Whitney *U* test. In this regard, the EDSS was considered an ordinal (non-continuous) variable.

Both the right and left foramina and the sum of their diameters were analysed. The foramen diameter in patients with MS was compared to that in control subjects. Similarly, a comparison was performed among patients with different MS disease courses. Bivariate correlation between the foramen diameter and the EDSS was estimated by calculating the Spearman correlation coefficient.

Intra-reader and inter-reader reproducibility were estimated using the Bland–Altman method. Considering the differences between the compared data sets, we calculated the coefficient of repeatability as 2 × standard deviation. Percent variability was calculated as the ratio of the coefficient of repeatability to the grand mean. Finally, the complement to 100% of percent variability was used as a measure of reproducibility [5]. Analyses were performed using SPSS Statistics (SPSS inc. v.20, Chicago, IL, USA). *P*-values <0.050 were considered statistically significant.

## Results

Out of 53 patients with MS, 39 (73%) had relapsing–remitting MS, 11 (21%) secondary progressive MS, 2 (4%) primary progressive MS, and 1 (2%) had a form of MS that was not clinically defined. The distribution of all continuous variables was normal (*p* ≥ 0.056), except for that of the left foramen in patients with MS (*p* = 0.001). Age at diagnosis was 33 ± 8 years. The median EDSS was 2.0 (interquartile range 1.5–4.5) (Table 1).

Data on the comparison of foramen diameter in patients with MS and controls are shown in Table 2. In particular, the mean diameter of the right foramen in MS patients was 10% smaller than that in controls; the mean diameter of the left foramen in MS patients was 7% smaller than that in controls; the sum of the diameters in MS patients was 8% smaller than that in controls.

**Table 2:** Comparison of the foramen diameter in patients with multiple sclerosis and control subjects

	Foramen diameter		
	Right	Left	Sum
Patients with multiple sclerosis (mm)	6.3 ± 1.6	5.6 ± 1.3	11.9 ± 2.3
Controls (mm)	7.0 ± 1.4	6.0 ± 1.3	13.0 ± 2.1
*p* value*	0.020	0.089	0.009

Data are means ± standard deviation

*Student *t* test

Data on the comparison between patients with relapsing–remitting MS and those with secondary progressive MS are shown in Table 3. The difference in foramen diameter was not significant (*p* ≥ 0.332). There was no significant correlation between the left (*r* = −0.242, *p* = 0.143) or right (*r* = −0.089, *p* = 0.595) foramen diameter and the EDSS, though there was a borderline significant correlation when the diameters of the right and left foramina were summed (*r* = −0.288, *p* = 0.079).

**Table 3:** Comparison of the foramen diameter in patients with multiple sclerosis in different courses of disease

	Foramen diameter		
	Right	Left	Sum
Relapsing-remitting (mm)	6.5 ± 1.7	5.6 ± 1.4	12.1 ± 2.4
Secondary progressive (mm)	6.2 ± 1.5	5.2 ± 1.1	11.3 ± 2.2
*p* value*	0.577	0.332	0.344

Data represent mean ± standard deviation

*Student *t* test

**Fig. 2:**
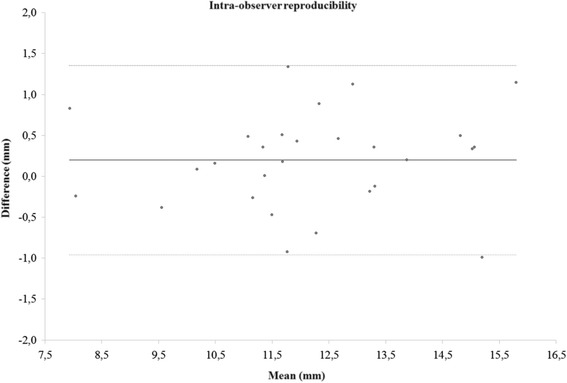
Bland–Altman graph showing intra-observer reproducibility for the measurement of the sum of diameters of both jugular foramina. Intra-observer reproducibility was 91%

**Fig. 3:**
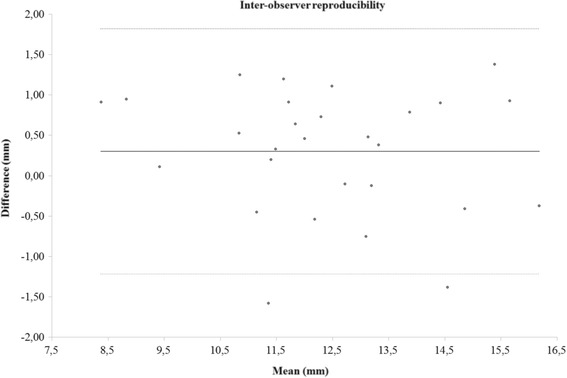
Bland–Altman graph showing inter-observer reproducibility for the measurement of the sum of diameters of both jugular foramina. Inter-observer reproducibility was 88%

Intra-reader and inter-reader reproducibility for the measurement of foramen diameter was 91% and 88%, respectively (Figs. 2 and 3).

## Discussion

Anatomical variants of jugular and vertebral venous systems have not yet been systematically investigated and classified. In recent years, several studies have reported vascular abnormalities in patients with MS. Zamboni and colleagues described an association between MS and reduced venous blood drainage of the central nervous system, a condition which is referred to as chronic cerebrospinal venous insufficiency (CCSVI) [6]. Although CCSVI has received attention in the MS community, the impact of CCSVI on venous cerebral outflow circulation and on the pathogenesis of MS has not been demonstrated. Indeed, the prevalence of CCSVI in patients with MS is highly debated, ranging from 0% to 100% [7–13].

Our preliminary results indicate that MS is associated with smaller jugular foramina diameter compared to control subjects. These data agree with a previously reported finding of an altered intracranial blood flow dynamics in patients with MS [3]. However, we cannot speculate on a causal relationship. On one hand, smaller jugular foramina can act as a stenosis on the cerebral blood outflow, reducing upstream cerebral perfusion; on the other hand, the opposite may be true, i.e. reduced cerebral perfusion may result in reduced downstream cerebral outflow with reduced jugular foramina size during cranial development. Further studies are necessary to demonstrate the impact of jugular foramina diameter on the overall cerebral blood flow and its potential role in the pathogenesis of MS. A small size of the jugular foramina could be an independent finding associated to the disease.

The clinical relevance of this study may only be speculated upon. Indeed, regardless of the actual relationship between foramen diameter and reduced downstream cerebral outflow, we may hypothesise that any endovascular venous angioplasty, as it is suggested for patients with MS [6, 10], might in any case be useless in patients with small jugular foramina. In fact, those treatments are typically performed in extracranial veins and a small jugular foramen would act similarly to an upstream stenosis.

We found no significant differences between patients with relapsing–remitting MS and those with secondary-progressive MS in terms of diameters of the jugular foramina, but this result may depend on the small sample size. Of note, we could not investigate this in patients with primary progressive MS due to the very limited number of those patients in our sample (two patients).

Similarly, there was no significant correlation between jugular foramen diameter and the EDSS, though there was a borderline significant negative correlation between the sum of the two diameters and the EDSS. Even though this correlation was only borderline significant, it is in line with the primary result of this study. In fact, it would suggest that the smaller the foramen diameters, the higher the EDSS, i.e. the worst the clinical status of the patients, which is a possibility to be confirmed by larger studies.

Apart from the limited sample size, a limitation of our study was that we measured the diameter of the venous lumen of the jugular foramina, rather than the osseous jugular foramina, which could probably be better evaluated with computed tomography. However, patients with MS are not followed up with cranial computed tomography, which is less sensitive for identification of lesions in MS and involves exposure to ionising radiation. However, the jugular vein is the functional structure deputed to blood flow drainage and occupies the largest portion of the jugular foramen. In addition, the reproducibility of our measurements was high even though the two readers had different levels of experience in brain MRI. Finally, we did not evaluate the impact on the results of confounders such as body height or body mass index.

In conclusion, our preliminary study showed that the diameter of the jugular foramina in patients with MS was 7-10% smaller than in controls, regardless of the clinical course of MS. Studies are warranted to confirm and explore the clinical relevance of this finding.

## Abbreviations

CCSVI: Chronic cerebrospinal venous insufficiency
EDSS: Expanded disability status scale
MRI: Magnetic resonance imaging
MS: Multiple sclerosis
